# The Roles of Embryonic Transcription Factor BRACHYURY in Tumorigenesis and Progression

**DOI:** 10.3389/fonc.2020.00961

**Published:** 2020-06-30

**Authors:** Ming Chen, Yinghui Wu, Hong Zhang, Suoyuan Li, Jundong Zhou, Jun Shen

**Affiliations:** ^1^Department of Orthopeadic Surgery, The Affiliated Suzhou Hospital of Nanjing Medical University, Suzhou, China; ^2^Department of Orthopeadic Surgery, Wuxi No. 2 People's Hospital, Nanjing Medical University, Wuxi, China; ^3^Suzhou Municipal Hospital, Suzhou, China; ^4^Suzhou Cancer Center Core Laboratory, The Affiliated Suzhou Hospital of Nanjing Medical University, Suzhou, China

**Keywords:** BRACHYURY, notochord, chordoma, tumorigenesis, transcriptional factor, EMT

## Abstract

Transcription factor brachyury, with a DNA-binding T-domain, regulates posterior mesoderm formation and notochord development through binding with highly conserved palindromic consensus sequence in a variety of organisms. The absence of brachyury expression in majority of adult normal tissues and exclusive tumor-specific expression provides the potential to be developed into a novel and promising diagnostic and therapeutic target in cancer. As a sensitive and specific marker in the diagnosis of chordoma, brachyury protein has been verified to involve in the process of carcinogenesis and progression of chordoma and several epithelial carcinomas in various studies, but the mechanism by which brachyury promotes tumor cells migrate, invade and metastasis still remains less clear. To this end, we attempt to summarize the literature on the upstream regulatory pathway of brachyury transcription and downstream controlling network by brachyury activation, all of which involve in both the embryonic development and tumor progression. We present the respective correlation of brachyury expression with tumor progression, distant metastasis, survival rate and prognosis in several types of tumor samples (including chordoma, lung cancer, breast carcinoma, and prostate cancer), and various brachyury gain-of-function and loss-of-function experiments are summarized to explore its specific role in respective tumor cell line *in vitro*. In addition, we also discuss another two programs relating to brachyury function: epithelial-to-mesenchymal transition (EMT) and cell cycle control, both of which implicate in the regulation of brachyury on biological behavior of tumor cells. This review will provide an overview of the function of master transcriptional factor brachyury, compare the similarities and differences of its role between embryonic development and carcinogenesis, and list the evidence on which brachyury-target therapies have the potential to help control advanced cancer populations.

## Introduction

The T-box genes encode a family of transcription factors, characterized by a highly conserved DNA-binding domain of about 180 amino acid residues, which is designated as T-domain (1, 2), and are essential in controlling many aspects of embryogenesis in a wide variety of organisms (3). Eighteen different mammalian T-box genes have been identified so far. T-box transcription factors preferentially bind to 24-nucleotide palindromic consensus sequence: AATTTCACACCTAGGTGTGAAATT (2).

The first of the T-Box family molecularly characterized is BRACHYURY (3, 4). *Brachyury* (“short tail” from Greek) origins from the phenotype of this gene mutant mice, most striking defect with a truncated tail, which was first described by Dobrovolskaia-Zavadskaia in 1927 (3). Orthologs of *Brachyury* have been identified in a large amount of multicellular organisms, such as ascidians, zebrafish, *Xenopus*, mouse, human, and others (4–6), which are required for posterior mesoderm formation and notochord differentiation, normal cell movements during gastrulation and tail outgrowth, and establishment of left–right asymmetry (4, 7). *BRACHYURY* encodes a protein of 435 amino acids, which functions as a transcription factor to bind with half site of abovementioned consensus sequence: TCACACCT.

Miettinen et al. (8) performed an immunohistochemical study of 5,229 cases, demonstrating nuclear BRACHYURY expression to be a sensitive and fairly specific marker for chordoma. Beyond that, BRACHYURY has been reported to express in various types of tumors (9–14), especially highly expressed in several tumors of epithelial origin. BRACHYURY expression is negative among most normal tissues, with the exception of testis and thyroid (15–17). The cause why BRACHYURY is absent in majority of adult non-neoplastic tissue and exclusively expressed in tumor-specific manner (18) drives researchers to discover the underlying role played by BRACHYURY on tumorigenesis.

## The Function of Brachyury in Mesoderm and Notochord Development

The *BRACHYURY (T)* gene is required for the formation of posterior mesoderm and axial development. In all vertebrates, the gene is initially expressed throughout the presumptive mesoderm, and during later stage, the expression is gradually restricted to the developing notochord and tail bud (3, 19, 20). Mutant embryos lacking *Brachyury* gene function demonstrate deficiency in notochord differentiation and the formation of posterior mesoderm but develop normal anterior mesoderm (3, 21). BRACHYURY expression is lost with maturation of the notochord, which disappears largely before birth. But some residual notochordal cells may persist in the intervertebral disks of the spine until early childhood and possibly throughout life in some people (15, 22).

Mice homozygous with *Brachyury* mutations will die shortly after gastrulation and display several mesodermal abnormalities (3, 4). *BRACHYURY* encodes sequence-specific activator that contains a T DNA-binding domain, through which BRACHYUYR exerts its mesoderm-inducing effects by directly activating downstream mesoderm-specific genes (4, 23). In addition, the role of *BRACHYURY* gene in developing mesoderm, morphogenesis, and cell fate is evolutionarily conserved (3).

## The Regulatory Network By Brachyury in Embryonic Development and Tumorigenesis

### Upstream Regulatory Pathway of *BRACHYURY* Transcription

Fibroblast growth factor (FGF) and fibroblast growth factor receptor (FGFR) signaling has been implicated in the patterning of mesoderm and activated *Brachyury* expression (24–28). In *Xenopus* embryos, the expression of *Xbra*, the *Xenopus* homolog of *Brachyury*, requires an intact FGF signaling pathway. Formation of mesoderm tissue requires a regulatory loop in which Xbra activates the expression of a member of the FGF family and FGF maintains the expression of Xbra (27, 29).

Another study in embryos of the ascidian found that *Brachyury* is expressed in a manner dependent on the FGF-mitogen-activated protein kinase kinase (MEK)-mitogen-activated protein kinase (MAPK)-Ets signaling pathway and on the intrinsic factors Zic and FoxA. Binding of Ets and ZicN at the 5′ upstream of *Brachyury* promoter region is required for FGF-responsive *Brachyury* gene activation in notochord precursor cells (30). In the chordoma cells, FGFR/MEK/extracellular signal-regulated kinase (ERK)/*BRACHYURY* pathway represents a novel therapeutic target (31). FGF2 induces MEK/ERK phosphorylation and upregulates *BRACHYURY* expression, *BRACHYURY* knockdown blocks the effects of FGF signaling, suggesting a positive feedback loop between FGF/FGFR and BRACHYURY could be required for chordoma cells' growth and survival.

The study by Hu et al. (32) suggests that FGFR1/MAPK signaling is also important for *BRACHYURY* activation in lung cancer cells. FGF1/FGFR1 signaling promotes ERK phosphorylation in the nucleus followed by transcriptional activation of *BRACHYURY*, which is further verified to be important for facilitating epithelial-to-mesenchymal transition (EMT), tumor cell growth, and invasion.

In addition, basic fibroblast growth factor (bFGF) has been reported to induce notochord formation and *Brachyury* expression in ascidian embryogenesis (26). Activin, BMP-4, Δp63, WNT3, WNT8A, BMP/Nodal pathway (33–37) have also been shown to regulate transcriptional activation of *Brachyury* in mouse, *Xenopus*, and zebrafish embryo and in tumor cells, human embryonic cardiomyocyte, etc.

### Downstream Regulatory Network by BRACHYURY Activation

BRACHYURY exerts its regulatory role by controlling transcription of a large number of target genes (23). Using ascidian *Ciona*, an invertebrate chordate, which is a commonly used model to study BRACHYURY function, over 50 validated genes have been identified to be controlled by BRACHYURY (38) In the embryo of *Ciona intestinalis*, Hotta et al. (20) showed that 20 of the putative BRACHYURY target genes encoded components for regulation of the cytoskeletal architecture, the extracellular matrix (ECM), proteins implicated in signal transduction and cell cycle control, etc. Morley et al. (7) investigated targets and gene regulatory network of No tail (Ntl), a zebrafish BRACHYURY ortholog, in mesoderm formation, discovering an *in vivo* binding site for Ntl, which accords with the conserved T-box binding site: TCACACCT. Ntl acts in combination with other factors, including other T-box factors and several signaling pathways, to mediate its activities in mesoderm development (7).

Further study by Katikala et al. (39) in 2012 revealed that transcriptional regulator BRACHYURY can establish multitiered transcriptional output and temporal readouts of target gene expression in ascidian Ciona. This molecule regulates most of its targets by directly activating early- and middle-onset genes, respectively, while indirectly controlling late-onset genes *via* transcriptional intermediaries.

The chief transcriptional targets of BRACHYURY in humans were firstly identified by Nelson et al. (40), integrating transcriptome data from chordoma U-CH1 cell line in which *BRACHYURY* was silenced with ChIP-seq data generated from the same cell line. Enriched gene sets controlled by BRACHYURY are mainly involved in the regulation of cell cycle and the production of ECM, multiple growth factors, and cytokines.

Yes-associated protein (YAP), an effector of the Hippo pathway and a master regulator of organ development (41), was recently found to be directly transactivated by BRACHYURY in chordoma cells through binding to the proximal region of the YAP promoter. Interestingly, BRACHYURY regulates YAP signaling through a non-transcriptional mechanism in lung carcinoma (18). Both BRACHYURY and YAP expressions were found to be elevated in glioblastoma and brain metastases originating from lung carcinomas, and *BRACHYURY* knockdown resulted in a significant decrease in YAP protein and mRNA expression in primary glioblastoma cells. BRACHYURY was identified as a positive regulator of YAP in various types of cancers (18).

## The Correlation of BRACHYURY With Clinical Tumors

### Chordoma

Although it is still unclear what role *BRACHYURY* could be in the tumorigenesis of chordoma, gene duplication mutation and overexpression in samples verified by previous various studies suggest that BRACHYURY might be a crucial molecular driver in the initiation and propagation of chordoma (42).

#### *BRACHYURY*/BRACHYURY expression in chordoma

Henderson et al. (43) performed a comprehensive study of the gene expression profile from 96 tumor samples with representatives of all mesenchymal tissues, *BRACHYURY* gene was found to be uniquely expressed in chordomas. By screening 53 chordomas, over 300 other neoplasms, and 33 normal tissues, BRACHYURY was found to be expressed in the embryonic notochord and all chordomas, labeling both chondroid and chordoid areas, and absent in all other neoplasms and non-neoplastic tissues (44). BRACHYURY is the first identified molecule to link notochord formation and chordoma pathogenesis (21). Miettinen et al. (8) immunohistochemically evaluated 5,229 different tumors for nuclear BRACHYURY expression, and all chordomas (75/76) were positive except a sarcomatous one. Another report (45) revealed that BRACHYURY was positively expressed in about 90% of all pathologically confirmed chordomas. As for exceptionally rare extra-axial skeletal chordomas and soft tissue chordomas, BRACHYURY was also reported to be a useful diagnostic tool (15, 46). All the above mentioned studies demonstrated that BRACHYURY expression (especial nuclear positivity) is a sensitive and fairly specific marker for the diagnosis and differential diagnosis of chordoma.

Our previous study on chordoma specimens inadvertently found two types of pathological components coexisting in the same one specimen, chordoma tumor elements with strong BRACHYURY expression and notochordal cell rests with rarely and no expression (47, 48). BRACHYURY was shown to be a sensitive (100%) and specific (100%) marker in distinguishing coexisting notochordal cell rests from chordoma tumor components (48).

#### The Role of *BRACHYURY*/BRACHYURY in Chordoma

JHC7 is the first chordoma cell line established with stable *BRACHYURY* expression. Silencing of *BRACHYURY* expression by using shRNA led to complete growth arrest and inability to be passaged serially *in vitro* (49). Similarly, U-CH1 cell line, which shows polysomy of chromosome 6 involving 6q27, was validated as representing chordoma by the generation of xenografts in the mouse model, demonstrating typical chordoma morphology and immunohistochemistry characteristics. Silencing of *BRACHYURY* in this cell line led to cell growth arrest and acquisition of a senescence-like phenotype (50).

#### The Genetic Basis of *BRACHYURY* Expression in Chordoma

Using combined genetic linkage and comparative genomic hybridization analyses, germline *BRACHYURY* duplication was identified to associate with the familial risk of developing chordoma (51), which is the first report of *BRACHYURY* copy number gain (CNG) in any disease type. Nevertheless, *BRACHYURY* duplication is extremely rare in sporadic chordoma (52).

Presneau et al. (50) and Dei Tos (53) demonstrated that close to half of the investigated chordoma cases showed a gain of chromosome band 6q27 (the locus wherein *BRACHYURY* locates) either through polysomy of the entire chromosome 6 or structural rearrangements, which indicates that cCNGs of *BRACHYURY* are pathogenetically relevant in sporadic chordoma. Cho et al. (35) and Pillay et al. (54) demonstrated that a common single-nucleotide polymorphism (SNP) located in the *BRACHYURY* gene, rs2305089, has strong association with the risk of sporadic chordoma.

In the following case-control comparison study (52), the risk estimated for rs2305089 was similar in familial and sporadic chordoma. Another common variant, rs1056048, was identified to strongly associate with familial chordoma with *BRACHYURY* duplication, and rs3816300 was significantly correlated with earlier age onset, which further corroborates the importance of genetic variations of *BRACHYURY* gene in the pathogenesis of both familial and sporadic chordoma. Recently, Sharifnia et al. (55) revealed that regulation of *BRACHYURY* by super-enhancers is a dominant feature of the chordoma gene-regulatory landscape. Chordoma JHC7 cells had a focal amplification at *BRACHYUYR* locus that encompassed proximal super-enhancers and a 1.5 Mb upstream region with broad H3K27ac occupancy, and patient-derived chordoma tumors were also found to have this hyper-acetylated region.

### Lung Carcinoma

Various studies have demonstrated that BRACHYURY is positively associated with the motility and invasiveness ability of lung tumor cell *in vitro* and highly expressed in late-stage lung tumor tissue, which supports *BRACHYURY* could be developed into a potential therapeutic target (12, 17, 56).

In addition, 37.5–62.5% of human lung cancer tissues are positive for *BRACHYURY* mRNA expression, which has a significantly higher percentage than normal lung tissue with 12.5% (10, 17). Moreover, BRACHYURY expression is significantly positively correlated with tumor stage (10) and no obvious relationship with histological type (12). High *BRACHYURY* mRNA expression significantly correlates with poor prognosis in both 5 year disease-free survival (DFS) and overall survival rate in primary lung carcinoma samples (12).

BRACHYURY protein expression was detected in ~41–60% of primary lung carcinoma tissues (17, 57) and 40% of non-small-cell lung carcinomas (NSCLCs) (16, 17), all of which demonstrated intense nuclear staining and weak, more diffuse cytoplasmic staining. BRACHYURY protein expression in the nuclei is significantly related to its mRNA level expression in lung cancer tissues (12). High expression of BRACHYURY protein is significantly associated with poor prognosis in overall survival (58) and high tumor stages, as well as lymph node metastases (59) in NSCLC samples.

*BRACHYURY*/BRACHYURY has been proved to play an important role in promoting lung tumor cell progression and metastasis *in vitro* (56). Seventy percent of lung cancer cell lines are positive for *BRACHYURY* mRNA expression (16, 17). *BRACHYURY*-inhibited lung H460 (10) and A549 cells (59) showed significantly reduced migratory and invasive capability. In addition, inhibition of *BRACHYURY* in H460 cells resulted in diminished capability to invade ECM and reduced expression of genes encoding for matrix metalloproteinase (MMP)2 and MMP24 (10), both of which participate in the ECM degradation. *BRACHYURY* expression did not influence primary tumor growth, whereas inhibition of *BRACHYURY* expression significantly diminished the ability of lung H460 cells developing experimental lung metastasis *in vivo*, whether by subcutaneous injection or by intravenous injection (10). All the above results suggest BRACHYURY is involved in several key steps of metastatic process in lung cancer cells: invasion, migration, adhesion, and colonization in the target organ.

*BRACHYURY* confers survival advantage to the lung cancer cells in response to treatment with various doses of the epidermal growth factor receptor (EGFR) inhibitor (17). Silencing of *BRACHYURY* in A549 cells increases cell sensitivity to cisplatin (59).

### Breast Carcinoma

*BRACHYURY*/BRACHYURY expression has been reported to positively associate with the invasive and metastatic capability of breast carcinoma cells *in vitro* and with the risk of recurrence and distal metastasis in breast patients (9, 60). Different studies have demonstrated the potential of *BRACHYURY* as a target for the treatment of breast carcinoma using cancer vaccines or immunotherapy approaches (61, 62).

*BRACHYURY*/BRACHYURY is obviously highly expressed at the mRNA and protein levels in breast cancer tissues and cell lines compared to negativity in normal breast cancer tissues and cells (9, 60, 63–66). Hormone receptor status of breast cancer is an important and recognized prognostic factor and can reflect different stages (67), including estrogen receptor (ER) and progesterone receptor (PR). *BRACHYURY* mRNA level expression in breast carcinomas with negativity for ER and/or PR is statistically significantly higher than those with positivity for ER and/or PR (9, 61), and triple-negative breast cancer (TNBC) is significantly higher than triple-positive and non-TNBC (61). Immunohistochemistry detection showed 90% of primary infiltrating ductal carcinomas were positive for BRACHYURY expression, comparing with almost absence of BRACHYURY in benign breast lesions. No significant differences were found between BRACHYURY protein level and various clinical parameters (grade, lymph node status, et al.) (9). Primary and metastatic TNBC samples showed 92–100% positive BRACHYURY protein expression, contrasting with <1% positive expression in adjacent normal breast tissue (61). Nuclear BRACHYURY protein expression is significantly higher in tumors of advanced stages III–IV than that of stages I–II (61) and an independent prognostic factor for DFS, while BRACHYURY cytoplasmic expression has no correlation with prognosis (68).

*BRACHYURY* gain-of-function and loss-of-function experiments were performed in various studies to investigate its role in breast carcinoma tumorigenesis, progression, and resistance to therapeutic intervention *in vitro*. Silencing of *BRACHYURY* in breast MDA-MB-436 cells statistically significantly reduced the ability to invade the ECM and form mammospheres in primary and secondary cultures (9). Our study (60) demonstrated that *BRACHYURY* promoted breast cancer cell invasion, migration, adhesion, and colonization in bone microenvironment *in vitro*, and *BRACHYURY* knockdown in MDA-MB-231 cells decreased the colonization and survival capability in bone tissue *in vivo*. BRACHYURY-high breast tumor cells were more resistant to the cytotoxic effects of docetaxel *in vitro* (9). *BRACHYURY* has also been confirmed to enhance breast cancer cell survival capability in response to tamoxifen therapy, and *BRACHYURY* silencing demonstrated more sensitive and higher apoptosis than control group upon tamoxifen treatment (63). Collectively, all the results of *in vitro* assays indicate that *BRACHYURY*-targeting therapeutic approaches under clinical trials and laboratory could have the potential to help control advanced breast carcinomas and improve prognosis.

### Prostate Cancer

BRACHYURY was shown to express in prostate cancer tissues, which increased with tumor malignancy and aggressiveness and was positively associated with Gleason score and TNM stage (69). Besides, *BRACHYURY*/BRACHYURY was also associated with tumor chemotherapy resistance (70). Targeting *BRACHYURY* is becoming a promising therapeutic option for advanced and metastatic prostate cancer patients.

Pinto et al. (14) demonstrated BRACHYURY nuclear staining was present in a comparable positive rate in prostatic intraepithelial neoplasia (PIN) lesions and prostate cancer tissue, contrasting with 100% positivity for metastatic prostate cancer. BRACHYURY nuclear expression is highly associated with the occurrence of metastasis (14, 69). There is a strong correlation between BRACHYURY expression and well-established markers of prostate cancer progression, such as Bcl2, ETS-related gene (ERG), and phosphatase and tensin homolog (PTEN) loss (70). A high level of BRACHYURY is verified to associate with poor prognosis (4, 69, 71). In addition, prostate cell lines with endogenous *BRACHYURY*/BRACHYURY expression were demonstrated to be more resistant to docetaxel and cabazitaxel treatment than that with negative expression (70).

Androgen receptor (AR) is the mediator of androgen activity in normal and malignant prostate cells. The BRACHYURY protein level in the nucleus of primary prostate cancer cells is statistically associated with the presence of AR (70), and the enhanced AR expression in the nucleus may be activated by BRACHYURY protein (70). Moreover, a genome-wide analysis on AR in prostate cancer cells revealed that BRACHYURY binding motif is highly enriched in AR-bound promoter region (72), suggesting that BRACHYURY is involved in AR regulation on target.

Although androgen-targeted therapy demonstrates recognized a therapeutic benefit in advanced prostate cancer, following castration-resistant prostate cancer (CRPC) develops and tumor progression occurs due to the induced epithelial-to-mesenchymal plasticity (EMP) and neuroendocrine transdifferentiation (NEtD) programs by androgen deprivation (71, 72), the mechanism through which has yet to be elucidated. Overexpression of BRACHYURY is strongly associated with NEtD markers, including chromogranin A (CHGA) and synaptophysin (SYP) (70), and targeting *BRACHYURY*/BRACHYURY has become a potential promising option in such a tricky scenario. A phase I/II trial (NCT03493945) testing a BRACHYURY-targeted antitumor vaccine has been performed in metastatic CRPC recently (73).

Although the specific role of *BRACHYURY*/BRACHYURY on the tumorigenesis and progression of prostate cancer has been recognized, more details need to be further investigated and unveiled, for instance, the mechanism of *BRACHYURY* involved in NEtD, the biological significance of BRACHYURY binding with the regulatory elements of the marker genes (*AMACR, AR*) of prostate cancer.

### Colorectal Cancer

*BRACHYURY* mRNA expression was found to elevate in tumors of the small intestine and in the majority of cell lines derived from the colon (16). Nearly 90% of the colorectal adenocarcinomas were immunohistochemically positive for BRACHYURY expression (74), which is demonstrated as distinct nucleus staining or widespread cytoplasmic staining (74, 75). The heterogeneity of BRACHYURY distribution suggests that it may have region-specific functions (75). High BRACHYURY expression correlates significantly with higher tumor stage, grade, and lymph node metastasis. Early-stage colorectal cancer samples (Dukes A) with BRACHYURY expression showed a significantly decreased survival and poor prognosis, while no correlation was observed in later tumor stages (74).

### Oral Cancer

Immunohistochemical studies demonstrated that BRACHYURY was positively expressed in 71.0% of oral squamous cell carcinoma (OSCC), including cytoplasmic and nuclear staining (76). BRACHYURY expression in OSCC tissue is significantly associated with lymph node metastasis (76), distant metastasis, and Anneroth scores (77). High BRACHYURY expression is also significantly associated with decreased disease-specific survival and DFS in OSCC patients, which may represent a valuable prognostic marker of OSCC (76, 77).

## The Mechanism of Brachyury To Promote Tumor Progression

### Epithelial-to-Mesenchymal Transition

The process of EMT, converting immotile epithelial cells to migratory mesenchymal cells, is associated with enhancement of invasive and metastatic potential of tumor cells, as well as resistance to therapeutic interventions (78, 79). BRACHYURY has been identified as a driver of EMT in a wide variety of tumors, including lung cancer (59), breast cancer (66), prostate cancer (14), hepatocellular carcinoma (80), oral squamous cell carcinoma (76), adenoid cystic carcinoma (81), among others, which is responsible for the acquisition of mesenchymal-like phenotype and positively correlates with aggressive characteristics of tumor cells (9, 10, 14, 16, 81–83).

Some other mediators have been reported to be involved in tumor EMT process, such as Slug, Snail, MMPs, fibronectin, interleukin (IL)8, and transforming growth factor (TGF)-β1, among others. Various studies have attempted to investigate the correlation between the expressions of these genes and *BRACHYURY* (12, 84, 85). As mesenchymal markers, Snail and Slug have been shown to act as transcriptional repressors of E-cadherin expression during the EMT process (86, 87). Fernando et al. (10) reported BRACHYURY could directly bind to the T-box half-site consensus sequence (TCACACCT) located at the promoter of E-cadherin, resulting in silencing of E-cadherin expression. BRACHYURY can directly enhance the Snail and Slug expression, through which can indirectly repress E-cadherin expression in several types of lung carcinoma cell lines (10, 82). In chordoma cells, loss of *BRACHYURY* resulted in a significant decrease of Snail and Slug (31, 49), and the upregulation of Snail and Slug by FGF2 was blocked by *BRACHYURY* knockdown, suggesting that BRACHYURY plays a critical role in the direct regulation of Snail and Slug expressions and EMT process of chordoma (31). BRACHYURY can directly bind with the T-Box binding sites at the promoter of Snail and fibronectin in prostate cancer cells (70).

Wan et al. (11) firstly revealed that BRACHYURY upregulated MMP12 expression in lung NSCLC cells to promote tumor cell migration and invasion, and a potential T-box binding site was found in the promoter of MMP12. In addition, Slug and IL-8 expressions were positively correlated with *BRACHYURY* expression at mRNA and protein levels in primary and metastatic lung tumor tissues and associated with poor prognosis (12, 58). In TNBC MDA-MB-436 cell line, silencing of *BRACHYURY* resulted in diminished vimentin and fibronectin expression and increased epithelial ZO1 expression (61). In prostate cancer cells, *BRACHYURY* expression was associated with a decrease of the epithelial marker and increased expression of mesenchymal signature genes, as well as upregulation of the MMP14, MMP24 (14, 70).

### The Effect of BRACHYURY on Tumor Cell Proliferation and Cell Cycle

Regulating of cell cycle progression is another paramount mechanism to modulate tumor cell biological behavior (88). Various studies have reported divergent effects of BRACHYURY on cell proliferation. Some demonstrated BRACHYURY promoted tumor cell growth and proliferation *in vitro*, including chordoma, prostate cancer, colorectal cancer, adenoid cystic carcinoma, and breast carcinoma cells (14, 31, 40, 42, 49, 50, 60, 75, 81). Whereas, others showed that BRACHYURY inhibited tumor cell growth and proliferation, including breast carcinoma, lung, and colorectal cells (9, 10, 82). The lower proliferation rate may protect tumor cells from stressful conditions, such as nutrient deprivation and genotoxic injuries induced by radiation or chemotherapy (79), accordingly, attain a certain survival advantage. The reported divergent roles of BRACHYURY on cell proliferation in specific cell line, for instance, breast carcinoma cell lines, need to be further elucidated, whether it is cell type-dependent or context-dependent or other causes.

In regard to the mechanism by which BRACHYURY inhibits cell proliferation in lung carcinoma cells, Fernando et al. (10) has revealed BRACHYURY blocks the cell cycle progression likely at the G1-S transition through suppressing cyclin D1 expression and activity of cyclin/CDK complexes. Huang et al. (82) have reported BRACHYURY impairs cell cycle progression and reduces tumor cell proliferation by transcriptional silencing of P21, through directly binding with the T-box half-site binding sequence located at position −14 relative to the transcription initiation site in the promoter of P21.

## The Tumor Suppressor Role of BRACHYURY

Unlike the established oncogenic function in some types of solid tumors, BRACHYURY has been reported to play a tumor suppressor role in lung cancer (89) and glioma (90). Pinto et al. (90) recently reported that glioma patients with absence or low level of BRACHYURY were associated with tumor aggressiveness and poor survival. BRACHYURY could have different functions in tumorigenesis and progression depending on the cofactors and specific context.

## Conclusion

The T-box transcription factor BRACHYURY, which is required for mesoderm formation and notochord development, has been recognized as a sensitive and fairly specific marker for chordoma and reported to be expressed in various types of tumors, especially in tumors of epithelial origin (lung, breast, prostate, colorectal, oral, et al.). BRACHYURY promotes tumor metastasis through modulating the EMT process and regulating cell cycle and closely correlates with patient poor prognosis. *BRACHYURY*/BRACHYURY has become an attractive target in the study of tumorigenesis and therapy not only because multiple signaling pathways converge to activate its expression (10) but also it regulates a complex downstream network. With the development of several clinical trials of therapeutic cancer vaccine (62, 91, 92), *BRACHYURY*/BRACHYURY will become a potential paramount target to help control advanced cancer populations.

## Author Contributions

All authors listed have made a substantial, direct, and intellectual contribution to the work and approved it for publication.

## Conflict of Interest

The authors declare that the research was conducted in the absence of any commercial or financial relationships that could be construed as a potential conflict of interest.
